# A Case of Bilateral Renal Cancer in a Dialysis Patient With Enlarged ACDK Treated With Staged Laparoscopic Nephrectomy

**DOI:** 10.1002/iju5.70158

**Published:** 2026-03-06

**Authors:** Rintaro Yoshitake, Shigeyuki Watanabe, Nanaka Maeda, Yukihiro Nagatani, Taichi Sano

**Affiliations:** ^1^ Hikone Municipal Hospital Hikone Shiga Japan

**Keywords:** acquired cystic disease of the kidney, acquired cystic disease–associated renal cell carcinoma, end‐stage renal disease, laparoscopic radical nephrectomy

## Abstract

**Introduction:**

End‐stage renal disease (ESRD) patients have a high risk of Acquired Cystic Disease‐associated Renal Cell Carcinoma (ACD‐RCC) due to chronic inflammation and cystic renal atrophy. Although laparoscopic radical nephrectomy (LRN) is standard, massively enlarged kidneys may increase surgical difficulty.

**Case Presentation:**

We describe a long‐term dialysis patient with bilateral ACD‐RCC. Each kidney weighed over 1200 g. Despite the extreme size and cystic architecture, LRN was completed without conversion, transfusion, or major postoperative complications.

**Conclusion:**

This case demonstrates that LRN can be safely and effectively performed in ESRD patients even when ACD‐RCC involves unusually large kidneys.

## Keynote Message

We report a case of bilateral ACD‐RCC successfully managed with staged LRN. Despite extreme kidney enlargement exceeding 1200 g on each side, both procedures were completed laparoscopically without conversion or major complications. This case demonstrates that LRN can be safely performed even in massively enlarged Acquired Cystic Disease of the Kidney (ACDK) kidneys when careful surgical planning is applied.

## 1 Introduction

ACDK is common in long‐term dialysis, and ACD‐RCC is its most frequent malignant tumor. Radical nephrectomy is usually required, and LRN is widely used because it is less invasive. However, nephrectomy in end‐stage renal disease can be difficult, especially when kidneys are enlarged or highly cystic. Reports of very large ACD‐RCC kidneys managed with LRN remain limited. Here, we describe a rare case of bilateral giant ACD‐RCC successfully treated with LRN.

### 1.1 Patient Information

A 54‐year‐old man (height 177.3 cm, weight 89.5 kg, BMI 28.47) with a history of end‐stage renal disease, acute subdural hematoma, and lumbar spinal canal stenosis was referred to our urology department after screening non‐contrast CT at a previous institution revealed incidental bilateral renal masses. Hemodialysis had been initiated at the age of 36, and he was receiving maintenance dialysis at a local clinic.

### 1.2 Clinical Findings

Laboratory tests at the initial visit showed the following values: Alb 3.3 g/dL, Cre 12.49 mg/dL, UN 54 mg/dL, Na 142 mmol/L, K 5.3 mmol/L, Ca 8.83 mg/dL, IP 5.7 mg/dL, UA 6.4 mg/dL, CRP 0.09 mg/dL, WBC 5400/μL, Hb 11.3 g/dL, Ht 33.5%, and Plt 175 000/μL.

**FIGURE 1:**
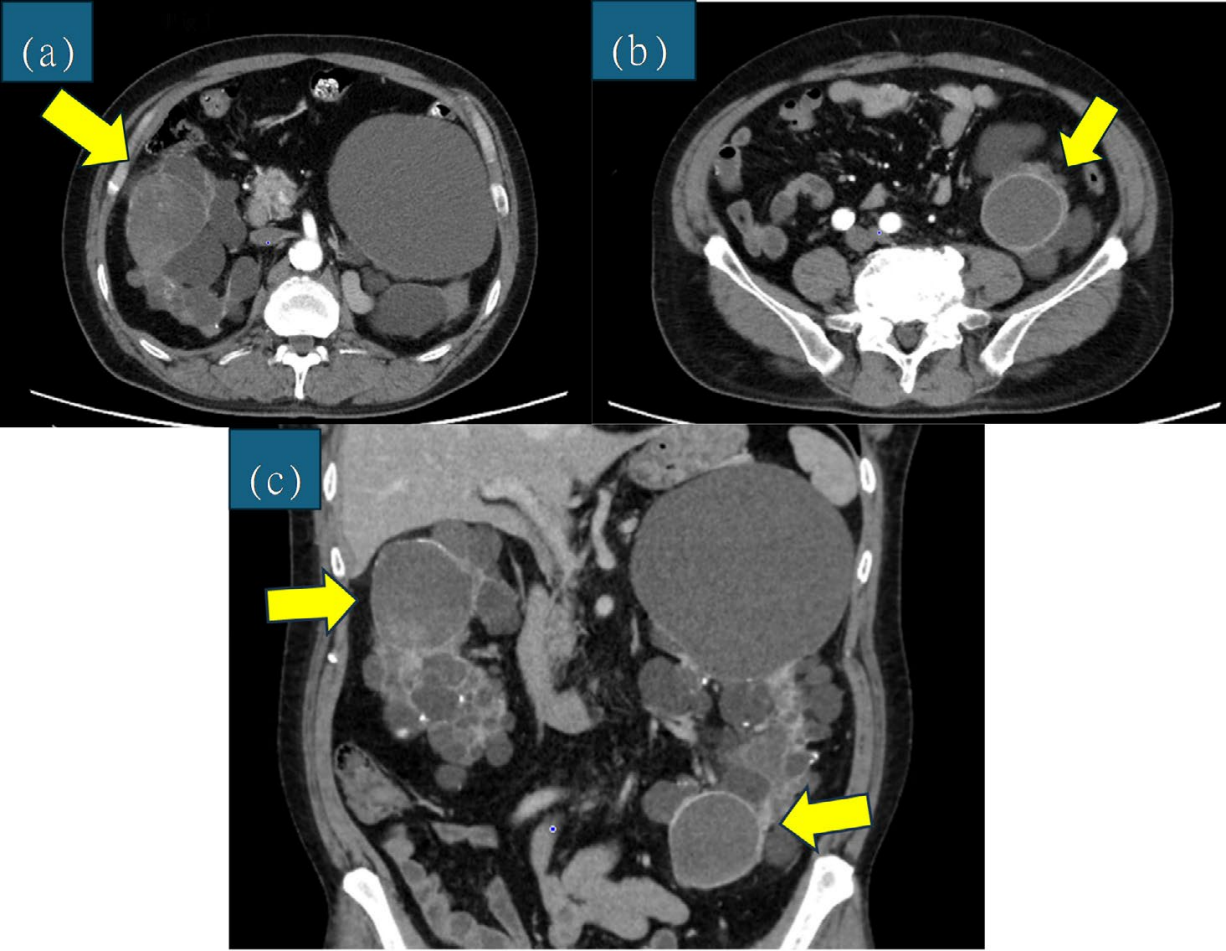
Contrast‐enhanced CT images. (a) Right kidney cancer, axial, (b) left kidney cancer, axial, (c) coronal. Cystic renal masses with a tendency to enlarge were observed in the mid‐pole of the right kidney and the lower pole of the left kidney, each containing an enhancing nodule. The maximal diameters were 64 and 49 mm, respectively. No obvious metastasis or venous invasion was detected. Right kidney dimensions: 133.5 mm (length) × 69.6 mm (width) × 146.3 mm (height). Left kidney dimensions: 170.0 mm (length) × 123.8 mm (width) × 234.3 mm (height).

Contrast‐enhanced CT demonstrated cystic renal masses in the right mid‐pole and left lower pole, each containing enhancing nodules (Figure 1a–c). Each tumor was solitary on both sides. The maximal tumor diameter was 64 mm on the right and 49 mm on the left. Based on these imaging findings, both tumors were diagnosed as renal cell carcinoma (cT1bN0M0). Although laparoscopic radical nephrectomy (LRN) was considered, both kidneys showed marked polycystic enlargement, with maximal kidney diameters of 15 cm on the right and 24 cm on the left. Because significant technical difficulty was anticipated, a staged surgical approach was selected.

### 1.3 Operative Course

**FIGURE 2:**
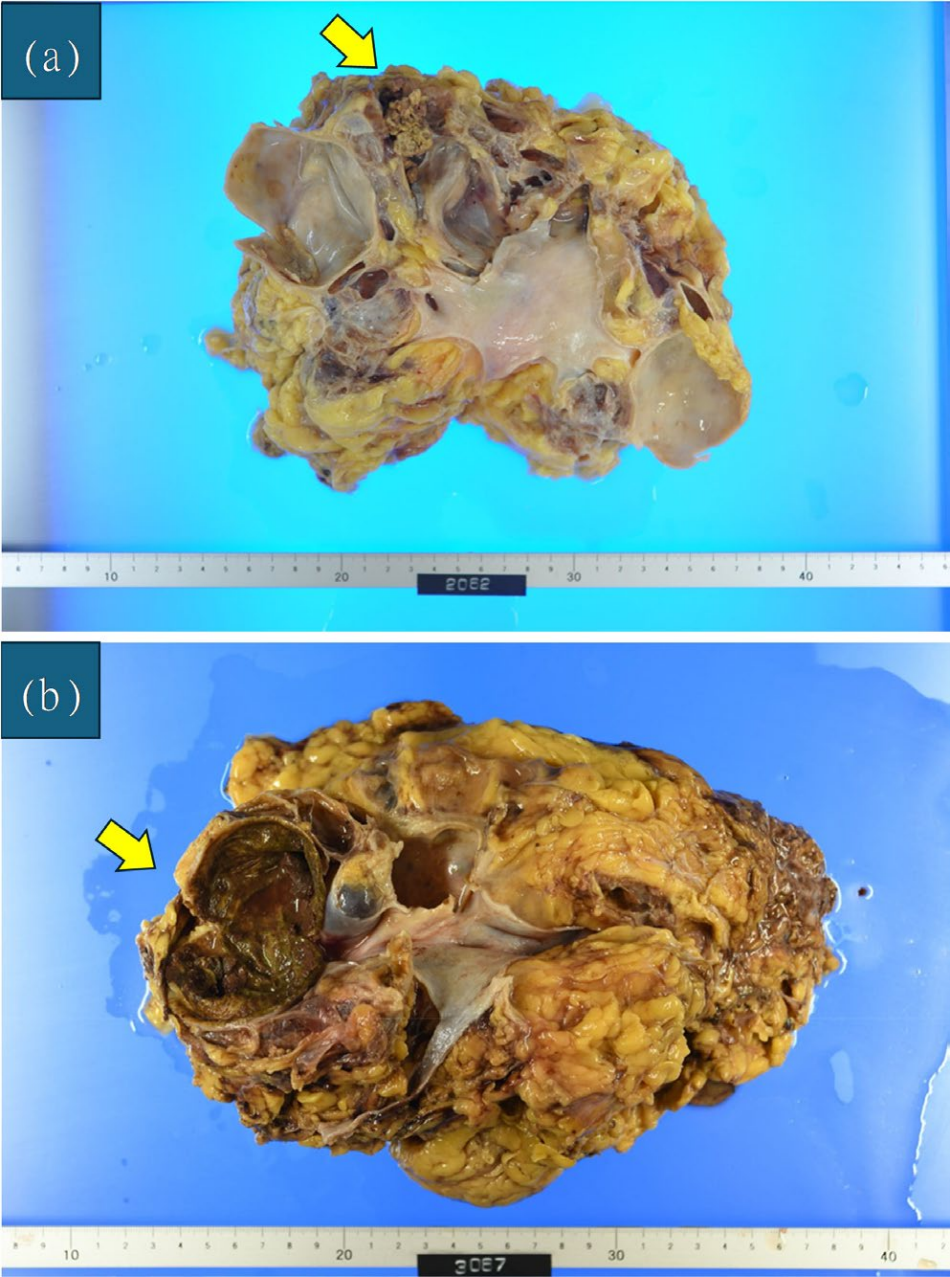
(a) Photograph of right kidney specimen. (b) Photograph of left kidney specimen. In both kidneys, a solitary tumor was identified against a background of atrophic, end‐stage acquired cystic kidney disease. Histopathological examination confirmed ACD‐RCC on both sides. The tumor measured 58 × 40 mm in the right kidney and 50 × 40 mm in the left kidney. Both tumors were pathologically staged as pT1b.

The first operation was performed 2 months after diagnosis. Right LRN via a retroperitoneal approach was completed with a pneumoperitoneum time of 5 h 15 min and a total operative time of 7 h 1 min. Blood loss was minimal. Because the specimen was too large to be accommodated in a retrieval bag, it was removed manually. For specimen extraction, the ventral right‐hand port site and the assistant port site were connected, and the skin incision was extended in the cranio‐caudal direction to a total length of approximately 12 cm. The resected specimen weighed 1265 g. The postoperative course was uneventful, and the patient was discharged on postoperative day 9. Pathological examination revealed pT1b “ACD‐RCC (Figure 2a)”.

**FIGURE 3:**
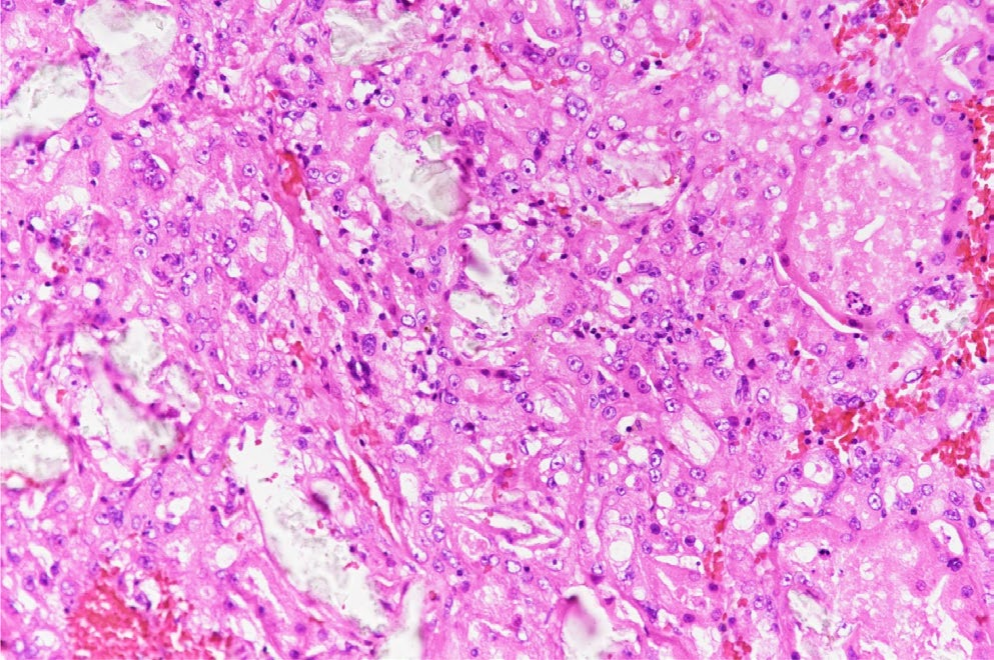
Histological examination of the left renal tumor reveals numerous microcysts and cribriform spaces, giving the lesion a “sieve‐like” appearance. Notably, several clear, refractile deposits consistent with calcium oxalate crystals are embedded within the tumor stroma and luminal spaces (arrows). These findings, occurring in the background of end‐stage renal disease and acquired cystic kidney disease, are pathognomonic for ACD‐RCC.

The second operation was performed 5 months after diagnosis. Left LRN using the same approach was carried out with a pneumoperitoneum time of 4 h 16 min and an operative time of 5 h 56 min. Blood loss was minimal. As with the right kidney, the ventral left‐hand port site and the assistant port site were connected, and the skin incision was extended in the cranio‐caudal direction to facilitate manual specimen extraction. The specimen weighed 1300 g, and the patient was discharged on postoperative day 11 without complications. Pathology again confirmed pT1b “ACD‐RCC (Figures 2b and 3)”.

Although both procedures required prolonged operative times due to difficulty securing an adequate surgical field, no major intraoperative or postoperative complications occurred. More than 3 years after surgery, the patient shows no evidence of recurrence or metastasis. His dry weight was adjusted according to the specimen weights, and maintenance hemodialysis continues uneventfully at his local clinic.

## 2 Discussion

According to the Kidney Data Registry of the Japanese Society for Dialysis Therapy (JSDT), a total of 343 508 patients were receiving dialysis in Japan at the end of 2023 [1]. Japan demonstrates markedly superior survival outcomes in hemodialysis care compared with Europe and the United States, with a crude 1‐year mortality rate of 6.6%, in contrast to 15.6% in Europe and 21.7% in the United States. After adjustment for age, sex, race, and 25 comorbid conditions, Japanese patients exhibited a 2.84‐fold lower relative mortality risk compared with European patients and a 3.78‐fold lower risk compared with American patients (both *p* < 0.0001) [2].

Patients undergoing dialysis are at an increased risk of developing kidney cancer compared with the general population, particularly malignancies of the kidney and urinary tract. Proposed mechanisms for this elevated risk include impaired immune surveillance, chronic inflammation, and the accumulation of carcinogenic metabolites, all of which contribute to heightened susceptibility to cancer in individuals receiving dialysis [3].

In dialysis patients, renal parenchymal atrophy frequently leads to the development of acquired cystic lesions, a condition defined as ACDK. Among malignant tumors arising in dialysis kidneys, ACD‐RCC is the most common, accounting for approximately 36% of cases [4]. ACD‐RCC is a distinct subtype of renal cell carcinoma that develops in end‐stage or ACDK kidneys and demonstrates characteristic morphological features. Grossly, it arises as a well‐circumscribed mass within an atrophic and extensively cystic renal parenchyma. Histologically, it displays diverse architectural patterns—including nested, tubular, multilocular cystic, and solid configurations—with hallmark sieve‐like or microcystic luminal structures observed within or between tumor cells [5].

Renal cell carcinoma in dialysis patients is generally detected at a low stage and low grade, and therefore is considered to have a relatively favorable prognosis [6]. However, spontaneous rupture has been reported in some cases [7], indicating the need for careful clinical monitoring.

Radical nephrectomy is considered the standard treatment for dialysis patients even in small renal tumors, as nephron‐sparing surgery offers no functional benefit in the setting of ESRD. Moreover, satellite tumors are present in approximately 30% of cases [8]. Laparoscopic radical nephrectomy (LRN) has become a major approach in contemporary radical nephrectomy due to its reduced invasiveness compared with open surgery. In our case, partial nephrectomy was deemed infeasible owing to the cystic nature of the tumor, and LRN was selected to minimize surgical morbidity. Furthermore, we selected a retroperitoneal approach because we were concerned that increased interference with intra‐abdominal organs might elevate the risk of postoperative complications such as ileus and intra‐abdominal adhesions. During surgery, special care was taken to avoid cyst rupture through gentle manipulation, and in the left nephrectomy, early aspiration of cystic fluid was performed to reduce kidney volume and facilitate dissection within the limited retroperitoneal space. As a result of these measures, the procedures were completed without any unexpected cyst rupture or related intraoperative complications.

Nephrectomy in patients with ESRD is associated with an increased risk of adverse outcomes, including a reported five‐fold higher in‐hospital mortality rate [9] and an elevated likelihood of requiring blood transfusion [10]. Conversely, Bird et al. [11] reported intraoperative and postoperative complication rates of 6.3% and 31.3%, respectively, in ESRD patients undergoing LRN, compared with 8.7% and 21.4% in non‐ESRD patients, with most complications being mild. Similarly, Yamashita et al. [12] demonstrated no significant differences in operative time or blood loss between ESRD and non‐ESRD patients undergoing LRN for RCC. These findings collectively suggest that LRN can be performed safely even in ESRD patients.

A literature search using PubMed with keywords including “ACDK,” “ESRD,” “dialysis,” “RCC,” and “LRN” identified studies that reported specimen weights (Table 1) [12, 13, 14, 15, 16, 17, 18]. Median specimen weights in these studies ranged from 296 to 883 g. Two studies included cases with specimen weights exceeding 1000 g; among them, Takagi et al. [17] noted that specimen weight was a significant predictor of conversion to open surgery, with all cases weighing more than 1170 g requiring conversion. In our case, the specimen weights were 1265 g on the right and 1300 g on the left, representing among the heaviest kidneys removed via LRN reported to date. Despite this, surgery was completed without conversion to open surgery, without transfusion, and without major complications.

**TABLE 1:** Summary of published reports of laparoscopic nephrectomy for patients with ESRD.

Author	Year	Patients/Renal units (*n*)	Approach (retroperitoneal/transperitoneal)	Operative time (min)	Bloos loss (mL)	Kidney weight (g)	P stage (I/II/III/IV)	Pathology	Hospital stay	Outcome
Barrett	1998	66/66	0/66	175 [100–292]	—	402.5 [115–964]	8/60/3/1	RCC etc.	4.4 [3–7]	No recurrence
Kato	2007	1/2	2/0	265 [245–280]	35 [0–70]	883 [800–966]	2/0/0/0	RCC, papillary carcinoma	—	No recurrence
Kim	2007	2/4	0/2	420 [360–480]	137 [120–154]	413.3 [261–628]	3/1/0/0	RCC, papillary carcinoma	6	—
Sanli	2010	17/20	16/4	119 [45–210]	81 [15–250]	317.6 [12–970]	—	—	2.7 [1–4]	—
Takagi	2011	117/139	129/10	249 [102–573]	30 [0–4320]	330 [52–2200]	118/7/7/7	RCC	—	2 recurrence; 1 died of a sepsis
Yamashita	2012	39/39	38/1	229 [105–431]	70 [10–660]	325 [90–1811]	—	RCC etc	8 [3–32]	—
Katsuoka	2020	2/2	2/0	—	—	296 [272–320]	2/0/0/0	ACD‐RCC, RCC	9.5 [7–12]	No recurrence
Our case	2025	1/2	2/0	388.5 [356–421]	Minimal	1282.5 [1265–1300]	2/0/0/0	ACD‐RCC	10 [9–11]	No recurrence

This study has several limitations. First, a substantial number of surgical cases involving dialysis‐associated renal cancer may remain unpublished, potentially introducing publication bias. Second, because ACD‐RCC was only incorporated into the WHO histological classification in 2016 [19], earlier reports may not have been captured in our literature search, limiting the completeness of available data.

## 3 Conclusion

LRN can be safely and effectively performed in ESRD patients even when ACD‐RCC involves unusually large kidneys.

## Ethics Statement

The authors have nothing to report.

## Consent

The authors have nothing to report.

## Conflicts of Interest

The authors declare no conflicts of interest.

## Data Availability

The data that support the findings of this study are available on request from the corresponding author. The data are not publicly available due to privacy or ethical restrictions.
